# THE IMPACT OF A NATIONWIDE PHYSICAL ACTIVITY INTERVENTION FOR DIABETES AND HYPERTENSION PREVENTION

**DOI:** 10.1093/geroni/igad104.1730

**Published:** 2023-12-21

**Authors:** Cynthia Chen, Gregory Ang, Chuen Seng Tan, Falk Müller-Riemenschneider, Yot Teerawattananon

**Affiliations:** National University of Singapore, Singapore, Singapore; National University of Singapore, Singapore, Singapore; National University of Singapore, Singapore, Singapore; National University of Singapore, Singapore, Singapore; Health Intervention and Technology Assessment Program, Muang Nonthaburi, Nonthaburi, Thailand

## Abstract

Increasing physical inactivity is a primary risk factor for diabetes and hypertension, contributing to rising healthcare expenditure and productivity losses. We examined the cost-effectiveness of the National Steps Challenge (NSC), an annual nationwide mHealth intervention to increase physical activity in Singapore. We used a Markov model to assess the long-term impact of increased physical activity from NSC on adults. Conducting NSC yearly for 10 years on a mean cohort size of 654,500 participants is projected to prevent 6,120 diabetes cases (95% credible interval: 3,690 to 9,040), 10,300 hypertension cases (6,260 to 14,700) hypertension cases and 4,950 death cases (3,280 to 7,040), leading to 78,800 QALYs (56,500 to 102,000) gained. From the health system perspective, assuming no differentiation of cost among different physical activity levels within each health state, the healthcare cost savings from the averted cases is estimated to be SGD674 million (239 million to 1.48 billion), with SGD364 million (57.8 million to 1.04 billion) for diabetes and SGD311 million (95.0 million to 690 million) for hypertension. Using a willingness to pay threshold of SGD10,000, NSC was cost-saving at -SGD4,510 (-14,000 to 1,430) per QALY gained. We project that increasing physical activity from a yearly nationwide physical activity intervention delays the incidence of diabetes and hypertension. Our results suggest that the intervention is cost-saving and improves the quality of life. The estimated cost savings are more significant when indirect costs are considered, hence providing important information for decision-making in countries when considering similar large-scale physical activity programmes.

